# Role of Frontal Functions in Executing Routine Sequential Tasks

**DOI:** 10.3389/fpsyg.2019.00169

**Published:** 2019-02-06

**Authors:** Chiharu Niki, Takatsune Kumada, Takashi Maruyama, Manabu Tamura, Yoshihiro Muragaki

**Affiliations:** ^1^Institute of Advanced Biomedical Engineering and Science, Tokyo Women's Medical University, Tokyo, Japan; ^2^Graduate School of Informatics, Kyoto University, Kyoto, Japan; ^3^Department of Neurosurgery, Neurological Institute, Tokyo Women's Medical University, Tokyo, Japan

**Keywords:** frontal lobe, executive function, routine sequential task, inhibition, action disorganization syndrome (ADS), action disinhibition syndrome (ADIS)

We voluntarily accomplish many routine tasks in daily lives, such as making tea and brushing our teeth. Most of our daily life is supported not by new, creative and innovative behaviors, but by routine, repetitive, and familiar sequential action tasks comprising of sequential action steps. For example, there are three steps to making a cup of tea, putting tea leaves into a teapot, pour hot water, and finally pouring the tea into a teacup. Cooper and Shallice (2000) noted that sequential actions include not only simple action schemas but “higher-level” sequential schemas, in terms of the decomposition of a goal into sub-goals for a sequential behavior. Similarly, in the morning, we face the higher-level sequential task of “preparing to go to work.” To accomplish this task, a person needs to perform sub-sequential routine tasks such as changing clothes, making breakfast, and eating breakfast, followed by brushing teeth and preparing and checking the briefcase. What are the brain functions that enable us to perform these sequential action tasks?

It is known that the frontal cortex is involved in intentional, goal-directed behaviors including action planning, monitoring, and decision-making, especially in the case of non-routine novel action tasks (Ridderinkhof et al., 2002; Rushworth et al., 2007; Anderson and Cui, 2009; Bhandari and Duncan, 2014). However, only a few studies have reported the contributions of frontal-executive functions in routine sequential action tasks (Luria, 1966; Schwartz, 1995; Humphreys and Forde, 1998). Theoretically, there are two different systems, contention scheduling (CS) that includes action schemas for executing routine tasks almost automatically, and the supervisory attentional system (SAS) that is involved in executing actions with attention (Norman and Shallice, 1980, 1986; Cooper, 2002). Furthermore, studies have suggested that SAS is supported by the prefrontal cortex (Burgess et al., 2000; Levine et al., 2000), whereas CS is related to the basal ganglia (Norman and Shallice, 1986) and the motor cortex (Rumiati et al., 2001).

Previous studies of patients with brain damage called “action disorganization syndrome (ADS)” have suggested several types of action errors in daily life and in experiments (Humphreys and Forde, 1998; Schwartz et al., 2002, 2003). For example, “step omission,” in which a patient fails to perform a necessary action step, and “step sequence,” in which a patient fails to follow a conventional order of action steps have been identified. Although individual patients showing these action errors had damage to their frontal lobes, the lesions suffered by the patients included not only frontal lobe damage, but also damage to posterior brain regions including the parietal and temporal lobes. Thus, it is possible that certain action errors mainly reflect damage to prefrontal functions, whereas other errors could reflect posterior-related dysfunctions.

Research on the frontal function in executing a routine sequential task has reported that patients with only the right frontal lobe damage suffer from the action disinhibition syndrome (ADIS, Niki et al., 2009). ADIS patients show only few step omission and step sequence errors which are frequently found in ADS patients, but on the other hand, they show many action errors of another type known as “distractor errors.” A distractor object is a non-essential object for accomplishing a target task. When a patient uses a distractor object, the action error is counted as a “distractor error.” For example, a set of objects used for calligraphy were presented with target objects for the task of “wrapping a gift.” In this condition, one of the patients with damage to the right frontal lobe wrote a list of gifts in calligraphy and then wrapped the gift. Writing a list in calligraphy was not very different from the purpose of the target task, and the behavior of writing a list of gifts in calligraphy is semantically related to the target task, which people that are familiar with calligraphy might do in an “everyday life” situation. However, in the test situation, none of the normal participants wrote a list when performing the identical task (Niki et al., 2009). It is possible that normal participants judged that tools of calligraphy were unnecessary for performing the target task of wrapping a gift. There are many objects in the world and it is important to decide which objects are necessary for a given situation. Patients that wrapped the gift after writing a list in calligraphy had to go through more steps than the normal participants and took longer to achieve the goal. Nevertheless, they seemingly did not mind taking more time. It has been reported that the right frontal lobe is related to the control of timing behavior (Picton et al., 2006, 2007; Vallesi et al., 2009). Therefore, it is possible that cognitive control for considering the optimal time for executing a sequential action is also impaired in these patients.

## Five Types of Action Errors Based on Brain Dysfunctions

Action errors of patients reported in previous studies could be divided according to possible cognitive dysfunctions. We summarized the types of action errors shown by individual patients in previous studies that described the lesion areas (Table 1). It seems that most action errors reflect not simple but multiple cognitive dysfunctions. Nevertheless, action errors found in sequential routine tasks could be divided into five types based on different brain dysfunctions: (1) Inhibition; (2) Monitoring and checking; (3) Selecting and matching action schema to objects; (4) Organization of action steps; and (5) Top-down control based on situational context.

**Table 1:** Types of cognitive impairments in performing routine sequential action tasks and related action errors and brain regions.

**Type of cognitive impairment**	**Type of action errors**	**Brain damaged regions in patients**
1 Inhibition	•Perseveration •Toying •Unrelated- distractor error	•R. frontal, inferior parietal lobule and angular gyrus (Humphreys and Forde, 1998; Forde and Humphreys, 2000) •R. frontal lobe (Niki et al., 2009)
2 Monitoring and Checking	•Incompleteness •Repetition	•R. frontal lobe (Niki et al., 2009)
3 Selecting and matching an action schema to an object	•Semantic •Addition •Tool omission •Quality and Spatial	•Bi. frontal lobe, anterior corpus callosum, bi temporal lobes (Schwartz et al., 1991) •Bi. temporal, L. inferior posterior parietal lobe, R. dorsolateral frontal cortex, orbitofrontal cortex (Schwartz et al., 1995) •Bi. medial and anterior temporal lobe, L. medial frontal cortex, L. superior parietal lobe, bilateral temporal and medial frontal lobes, L. inferior and medial frontal gyri (Bickerton et al., 2007) •L. occipito-temporal cortex (Morady and Humphreys, 2011) •L. frontal lobe (Humphreys and Forde, 1998) •L. fronto-temporo-parietal lobe (Rumiati et al., 2001)
4 Organization of action steps	•Sequence •Step omission	•Bi. Anterior inferior temporal cortex (Buxbaum et al., 1997) •Bi. medial and anterior temporal lobe, left medial frontal cortex, L. superior parietal lobe, bilateral temporal and medial frontal lobes, L. inferior and medial frontal gyri (Bickerton et al., 2007) •R. frontal, inferior parietal lobule and angular gyrus (Humphreys and Forde, 1998; Forde and Humphreys, 2000) •L. occipito-temporal cortex (Morady and Humphreys, 2011) •Bi. frontal lobe, anterior corpus callosum, bi temporal lobes (Schwartz et al., 1991) •Bi. frontal, temporal, occipital gyri (Humphreys and Forde, 1998; Morady and Humphreys, 2008) •L. frontal lobe (Humphreys and Forde, 1998) •RCVA (R. cerebral vascular accident) group, (Schwartz et al., 1999; Schwartz, 2006) •LCVA (L. cerebral vascular accident) group, (Schwartz, 2006)
5 Top-down control based on a context of situation	•Related- distractor errors	•R. frontal lobe (Niki et al., 2009)

It is possible that the first type of brain dysfunction, “inhibition,” is mainly associated with action errors such as “perseveration,” “toying,” and “unrelated-distractor errors.” In perseveration, a person repeats the same action step that was just completed. In toying, a person might, for example, hold and lift-up an object or fiddle with it, without actually using the object. In unrelated distractor errors, a person uses a distractor object that is unrelated to the target task. Different from “related distractor errors,” unrelated distractor errors are not semantically associated with the target task. An example of an unrelated distractor error is when the target task is wrapping-up a gift and the patient writes the word, tree, in calligraphy, instead of writing a list of gifts. These action errors reflect an impairment in the inhibition for unnecessary objects and performing unnecessary action steps.

The second type of brain dysfunction, monitoring, and checking is known to influence incomplete and repetition errors. Monitoring and checking is the function of monitoring an action and detecting action errors (Luu et al., 2000; Ridderinkhof et al., 2002). Incompleteness is an action error in which a participant does not complete an action step to the end, but instead, starts a different action. Each action step includes several sub-action steps. For example, the first step of making a cup of tea is putting tea leaves in a pot, which includes sub-steps such as opening the tea canister and spooning-up tea leaves. When a person opens a canister of tea but does not spoon-up the tea-leaves, it is considered an example of incompleteness. Then, the person needs to return to the same action step that was not completed. If the monitoring and checking system detects that a certain action step was incomplete, the person might deviate from the action step that was just conducted before finishing it. Moreover, repetition errors happen when a person inappropriately repeats an action step later in the sequence. Such errors might be prevented if the system could detect that a particular action step had already been completed and that it is unnecessary to repeat the step.

The third dysfunction is “selection and matching an action schema to an object.” This dysfunction is associated with “semantic,” “addition,” tool omission,” and “quality and spatial” errors. In the process of conventionally using objects in a routine task, conventional action schema related to the objects are selected and matched, instead of selecting and matching novel objects. If wrong action schema were selected and matched with objects, a patient would display semantic” errors. On the other hand, if not only one action schema but several action schemas were activated and allocated to an object, patients might show addition errors, in which a patient adds extra action steps to an object. For example, the person that rips a teabag open and pours loose tea-leaves into a teapot has conducted an addition (Humphreys and Forde, 1998). It has been reported that a patient with damage to only the left frontal lobe displayed no addition errors when only necessary objects were presented to him, whereas he showed a few “addition” errors when distractor objects that were semantically related to the target objects in the sequential routine task were also presented. In this case, Humphreys and Forde (1998) suggested the possibility of competition among action schemas. Thus, it is possible that when distractor objects are presented there is a conflict between action schemas, which is followed by selecting the correct action schema by the frontal lobes to resolve the conflict (Giovannetti et al., 2010). Tool omission is when a participant fails to use the correct object. This could be considered a failure in finding the correct action schema for an object (for example, spreading jelly with fingers instead of a knife, Schwartz et al., 1999). Moreover, quality and spatial errors refer to inappropriate degrees of using an object and spatial misuses of an object, such as cutting paper to a size that is too small for wrapping a gift (Schwartz et al., 1999). These errors could reflect control deficits in selecting or matching a correct action schema to an object.

The forth dysfunction, the “organization of action steps” could result in “sequence” and “step omission” errors. It has been suggested that organizing sequences of action steps are not impaired by damage to the frontal lobes (Humphreys and Forde, 1998) because routine sequential action tasks need little attentional control. Conversely, it could be the case that deficits in the organization of action step are observed under conditions in which attentional control is required. On the other hand, when sequential knowledge of action schema for a certain sequential task itself is impaired, a patient would also show sequence and step omission action errors. Thus, an impairment of organizing action steps might be observed after damage to attentional control areas of the brain as well as areas responsible for sequential knowledge or a combination of these two functions (Schwartz and Buxbaum, 1997). However, there might be some behavioral differences between sequence and step omission errors that are observed as a result of deficits in attentional control and sequential knowledge. Deficits in the organization of action steps might relay situational conditions, for example, whether a top-down attentional control is needed or not. Therefore, more detail investigations of action errors and related brain regions are required in the future.

Finally, the top-down control based on the situational context could result in “distractor errors related to a target task,” in which patients use distractor objects that are semantically related to the target task. Although normal participants never use distractor objects, patients with damage to the right frontal lobe use distractor objects that are related to a target task (Niki et al., 2009). The patients might implicitly or explicitly consider that distractor objects are needed to administrate a target task despite the instruction that they need not use all the objects that were provided to them. Importantly, the patients did not perform actions unrelated to the target task. Since the purpose of using distractor objects was matched to that of a target task, it is possible that action sequences of distractor objects semantically invades the action sequence of a target task. In other words, it could be that distractor action sequences were embedded into the target sequence at the planning stage.

Investigating real behaviors of non-patients performing routine sequential actions, such as script card sorting, has shown that patients with frontal lobe damage make unconventional semantic decisions regarding verbal action scripts (Sirigu et al., 1996). For example, a patient decided to use an “increase volume” script for a radio in a shampoo script and made a new script stream, “increase *hair* volume” (Sirigu et al., 1996). Moreover, it has been reported that patients with frontal lobe damage could not discard distractor scripts in a distractor script card sorting task, although normal participants could discard them. Furthermore, common sense for semantic frames of a familiar situation might be impaired after damage to the frontal lobe. In relation to common sense, it has been reported that the responses of patients with right frontal lobe damage to several typical situations, such as a wedding ceremony were not atypical, but were different from those of healthy participants and patients with left frontal lobe damage (Baldo et al., 2016). Typical verbal representations such as saying “congratulations” are common in weddings and graduation ceremonies. However, responses of patients with frontal lobe damage were unsuitable for these situations. Therefore, it is possible that top-down control and selection of suitable action steps based on the common semantic frame of a situation are required for making appropriate, situation-specific, and responses.

## What Should There be Control of Familiar Sequential Action Tasks? -The Role of the Frontal Lobes

Familiar sequential action tasks such as brushing our teeth or making a cup of tea are repeatedly performed in daily life, and the sequential knowledge of these routine actions has been stored and can be activated nearly automatically. When these familiar actions are conducted in daily life, or under experimental conditions, they must be controlled by frontal function such that they fit the situation at the specific time. As mentioned in Osiurak et al. (2010), executing a routine task in an experiment differs from that in real life. It has been suggested that the supervisory processes play an important frontal function in experimental situations, such as monitoring, guiding, and checking the information processed during every day sequential action tasks (Land et al., 1999; Cooper, 2002). However, will these frontal functions act identically in everyday life as in experimental situations? If you need to write a letter in daily life, it could be done while pouring tea if there were sufficient time. However, if there is no time to pour tea, planning the action steps of writing the letter must be arranged in the shortest appropriate action sequence to match situational demands. The frontal lobe accepts information about the context of situations and plans which action steps and action sequences are optimal for a given situation. Obviously, daily life and experimental situations are different. Nevertheless, there is no situation that does not include some situational context, even in an experiment. Therefore, planning an action-sequence for deciding which action steps are needed has no exceptions. Each sequential action step is guided by the frontal lobe from the beginning to the end (Humphreys and Forde, 1998), and the prefrontal lobes enable organizing adaptive behaviors to variable environments (Koechlin, 2016). Furthermore, it has been reported that planning impairments are associated with lesions to the right dorsolateral prefrontal cortex (Burgess et al., 2000). The attentional process of planning might always be necessary for performing daily, routine tasks in situations requiring us to fit our behavior to the context of a situation, although each related action schema of presented objects in the tasks could be activated nearly automatically (Tucker and Ellis, 1998; Grezes et al., 2003). Moreover, the actual objects that are used and how these objects are used depends on the action plan. The results of using distractor objects, especially using distractor objects related to a target task by ADIS patients with damage to the frontal cortex might reflect inhibitive dysfunctions and impairments of the planning function. Normal subjects might not use distractor objects because they do not plan action sequences including the use of distractor objects. It has been reported that environmental adaptation such as the array of required objects, reduced action errors in everyday tasks, suggesting the need for assistance from the environment in reducing planning demands of the task (Kessler et al., 2015). ADIS patients did not show any expressions that something was strange or any perplexity even when using distractor objects because they were only performing sequential tasks according to their own plan. These patients used distractor objects voluntarily. Thus, the free will for executing familiar, sequential tasks might be controlled by planning in the prefrontal cortex, although the actual process of planning is done almost automatically and implicitly.

In this opinion, deficits in routine familiar sequential tasks observed in patients with brain damage and related impairments of brain function were discussed. It is suggested that the frontal lobes are related not only to performing novel tasks but also to performing routine sequential tasks according to different situational demands. Future, experiments on sequential routine tasks with controls for several different conditions might reveal more details about the frontal functions of executing routine sequential tasks. The instructions given to participants of such experiments and objects that are presented to participants could result in several kinds of contextual demands. Moreover, there is no situation that does not present contextual information regarding the situation, even under experimental conditions. Therefore, it is important to control for these conditions when investigating the behaviors of patients with frontal lobe damage. It seems that the frontal lobe subtly controls action sequences.

## Author Contributions

CN and TK designed and conducted the research, analyzed the data, and wrote the paper. TM, MT, and YM arranged the patients and identified the regions of brain damage. All authors have made an intellectual contribution to the work and approved it for publication.

### Conflict of Interest Statement

The authors declare that the research was conducted in the absence of any commercial or financial relationships that could be construed as a potential conflict of interest.
